# Occurrence and Genetic Diversity of *Cryptosporidium* spp. in Pet Rodents from Yunnan, China: Identification of Zoonotic Subtypes in Hamsters

**DOI:** 10.3390/ani16081177

**Published:** 2026-04-12

**Authors:** Liujia Li, Xinjie Yang, Muhammad Sohail Sajid, Yongyi Wang, Ze Li, Qin Xie, Luyang Wang, Junjun He, Fengcai Zou, Fanfan Shu

**Affiliations:** 1 The Yunnan Key Laboratory of Veterinary Etiological Biology, College of Veterinary Medicine, Yunnan Agricultural University, Kunming 650201, China; liliujia2007@163.com (L.L.); yangxinjiee@sina.com (X.Y.); fan19829810607@163.com (Y.W.); lz20041110@outlook.com (Z.L.); 15126606541@139.com (Q.X.); wly952659965@foxmail.com (L.W.); hejunjun617@163.com (J.H.); 2 College of Agriculture and Biological Science, Dali University, Dali 671003, China; 3 Department of Parasitology, University of Agriculture, Faisalabad 38000, Pakistan; drsohailuaf@uaf.edu.pk

**Keywords:** *Cryptosporidium* spp., pet rodents, occurrence, zoonosis, *SSU* rRNA gene, *gp60*, MLST, Yunnan province

## Abstract

*Cryptosporidium* spp. are common protist pathogens, and the growing popularity of pet rodents raises concerns about their potential role in zoonotic parasites transmission. However, epidemiological data on *Cryptosporidium* spp. in pet rodents in Yunnan Province is scarce. To examine the occurrence of *Cryptosporidium* spp. in pet rodents in Yunnan, we collected 762 fecal samples from four rodent species across four cities. Nested PCR and DNA sequencing were used to characterize the species and subtypes of *Cryptosporidium*. The occurrence of *Cryptosporidium* spp. in guinea pigs (*Cavia porcellus*), Siberian dwarf hamsters (*Phodopus sungorus*), Syrian hamsters (*Mesocricetus auratus*) and fancy rats (*Rattus norvegicus domestica*) was 18.7% (80/426), 17.3% (36/207), 12.5% (15/120), 0% (0/9), respectively, with an overall rate of 17.2% (131/762). According to regions, the positivity rate of *Cryptosporidium* spp. in Zhaotong city, Kunming city, Yuxi city and Qujing city was 21.0%, 17.9%, 16.8% and 10.5%, respectively. In terms of sampling location, the occurrence of *Cryptosporidium* spp. in pet markets, farms and shops was 19.5%, 18.6% and 0%, respectively. Sequence analysis of the small subunit ribosomal RNA (*SSU* rRNA) gene identified six *Cryptosporidium* species/genotypes: *Cryptosporidium homai* (*n* = 52), *Cryptosporidium wrairi* (*n* = 30), *Cryptosporidium* sp. hamster genotype (*n* = 25), *Cryptosporidium andersoni* (*n* = 20), *Cryptosporidium parvum* (*n* = 5), and *Cryptosporidium muris* (*n* = 1). Further subtyping of *C. andersoni* isolates using multilocus sequence typing (MLST) revealed a single subtype, with all isolates identified as A3A4A2A2. All five *C. parvum* isolates were identified as subtype IIdA15G1 based on the *gp60* gene. Our findings demonstrated the presence of the zoonotic *C. parvum* IIdA15G1 subtype in pet rodents, suggesting that these animals, particularly hamsters, may serve as reservoirs for human-pathogenic *Cryptosporidium* species. These results underscore the need for improved biosecurity and husbandry practices in the pet rodent trade to mitigate public health risks.

## Simple Summary

*Cryptosporidium* spp. cause diarrhea in animals and humans. With the increasing popularity of pet rodents, their potential role in transmitting these parasites requires closer attention, particularly in regions like Yunnan, China, where such data are limited. In this study, we examined fecal samples from 762 pet rodents in four cities in Yunnan Province, China, to determine the presence of *Cryptosporidium* and subtype identification. Overall, 17.2% of the animals tested positive for the parasite. Six different *Cryptosporidium* species were identified, including *C. parvum* subtype IIdA15G1. This zoonotic subtype was found in pet hamsters. Rodents from pet markets and breeding farms had higher positivity rates than those from pet shops, suggesting that high-density housing conditions and poor hygiene increase the risk of infection in animals. Our findings suggest that pet rodents, particularly hamsters, can carry *Cryptosporidium* spp. that infect humans, underscoring the importance of improved husbandry practices and public education to safeguard animal and human health.

## 1. Introduction

*Cryptosporidium* spp. are globally distributed apicomplexan parasites that cause cryptosporidiosis, a major diarrheal disease affecting humans, livestock and companion animals [1]. They are a leading cause of food- and water-borne outbreaks of gastroenteritis worldwide [2]. In humans, cryptosporidiosis is particularly severe in young children and immunocompromised individuals, and it remains a significant contributor to diarrhea-related morbidity and related mortality [3]. Transmission primarily occurs via the fecal–oral route, through direct contact with infected animals or exposure to contaminated environment [4]. Due to the broad host range of *Cryptosporidium* spp., zoonotic transmission is recognized as a critical factor in the epidemiology of human cryptosporidiosis [5].

To date, over 48 valid *Cryptosporidium* species and approximately 120 genotypes have been identified [6]. Of these, at least 28 *Cryptosporidium* species and 41 genotypes have been reported in rodents [7,8,9]. Importantly, approximately 20 of these species are recognized as zoonotic pathogens, posing a direct public health risk [9,10]. This highlights the potential public health risks associated with rodent-borne *Cryptosporidium*. Molecular characterization is essential for accurately identifying *Cryptosporidium* species/genotypes and assessing their zoonotic potential [11], and several genetic typing tools have been developed for this purpose [12]. The most widely used method for distinguishing *Cryptosporidium* spp. is the 60-kDa glycoprotein (*gp60*) gene subtyping method, which contains a microsatellite/minisatellite region consisting of poly-serine-encoding trinucleotide repeats (TCA, TCG and TCT) [13]. The MLST method, which targets four minisatellite/microsatellite loci (MS1, MS2, MS3, and MS16), is commonly used to evaluate the genetic diversity and population structure of species such as *C. andersoni* [14,15].

As the largest order of mammals, rodents are considered one of the primary reservoirs for *Cryptosporidium* spp. [16]. Among the different rodent populations, pet rodents exhibited the highest prevalence at 27.0%, followed by wild rodents at 20.5%, farm rodents at 14.5%, and finally laboratory rodents with the lowest rate at 2.7% [9]. Due to their large population, wide distribution, and ground-level activities that facilitate contact with *Cryptosporidium* oocysts, rodents have frequently tested positive for this pathogen [17]. Pet rodents, owing to their proximity to humans, are recognized as potential vectors for zoonotic pathogen transmission [18]. In modern life, rodents are increasingly popular pets due to their cute appearance, gentle nature and easy care [17]. Despite their growing popularity as pets in China and their potential role as reservoirs for zoonotic pathogens, epidemiological data on the prevalence, species distribution, genetic diversity and zoonotic significance of *Cryptosporidium* spp. in pet rodents is largely lacking.

Yunnan Province is characterized by its biodiversity and humid subtropical climate. These characteristics provide an environment conducive to the transmission of enteric parasites [19]. The province raises a large number of companion animals, including pet rodents. However, little is known about *Cryptosporidium* infections in pet rodents in Yunnan. Previously, only one small-scale study has documented *Cryptosporidium* infections in wild rodents in the province [20]. Thus, this study aimed to investigate the occurrence, genetic diversity and zoonotic potential of *Cryptosporidium* spp. in pet rodents in Yunnan to evaluate their public health significance.

## 2. Materials and Methods

### 2.1. Sample Collection

**Figure 1:**
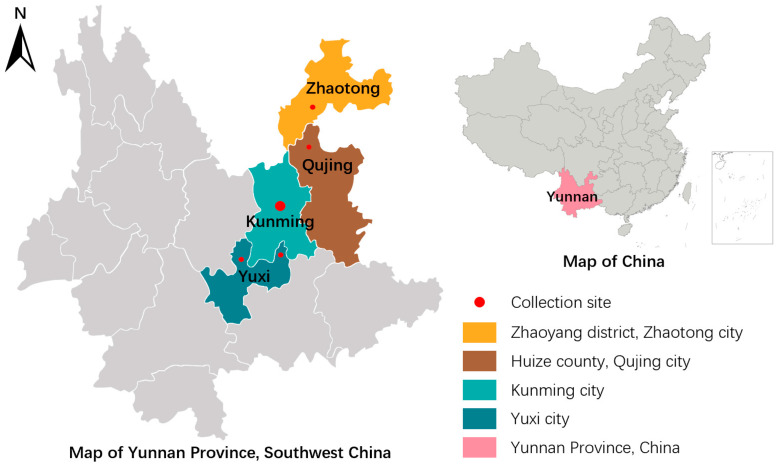
Sampling sites in Yunnan Province, China, for the study of *Cryptosporidium* spp. in pet rodents.

From October 2024 to December 2025, a total of 762 fecal samples from four species of rodents were collected across three pet stores, eight pet markets, and five pet breeding farms in Yunnan Province (Table 1 and Figure 1).

Of the three pet stores located in Kunming, 69 samples were collected from Syrian hamsters (*Mesocricetus auratus*) and four were collected from fancy rats (*Rattus norvegicus domestica*). The rodents were housed individually, and one fresh sample was collected per cage. All pet stores followed a daily cleaning protocol that included disinfecting cages with alcohol spray and replenishing food and bedding every morning. From the eight pet markets distributed across Zhaotong, Qujing, and Kunming, 137 samples were collected from Siberian dwarf hamsters (*Phodopus sungorus*), 78 from guinea pigs (*Cavia porcellus*), 51 from Syrian hamsters, and five from fancy rats. Each animal was captured by hand from its cage, and fecal samples were collected after it defecated spontaneously. Additionally, these markets sold other pets, such as dogs, cats, and birds. The markets did not implement daily disinfection or bedding replacement for rodent enclosures. A total of 418 samples were collected from the five breeding farms: two in Yuxi (one breeding guinea pigs and one breeding Siberian dwarf hamsters) and three in Kunming (all breeding guinea pigs). The guinea pigs were housed in groups of 10–30 per pen, while the hamsters were housed in groups of 50–100 per container. Three to four individual rodents were randomly captured from each pan and fresh fecal samples were collected from each selected animal.

None of the examined rodents had received antiparasitic treatment prior to sampling, and no diarrhea or other clinical symptoms were observed. Fecal samples were manually collected using disposable gloves and placed in sealed bags. The bags were labeled with information including the collection date, location, animal species, coat color, and clinical notes. All samples were preserved in a 2.5% potassium dichromate solution at 4 °C before DNA extraction.

### 2.2. DNA Extraction and PCR Amplification

Prior to genomic DNA extraction, each fecal sample was washed twice with distilled water and centrifuged at 2000× *g* for 10 min at room temperature to remove potassium dichromate. Approximately 200 mg of the washed fecal material was then used to extract the genomic DNA with the E.Z.N.A.^®^ Stool DNA Kit (Omega Bio-Tek, Norcross, GA, USA), following the manufacturer’s instructions [21]. The DNA was eluted in 100 μL of elution buffer and stored at −20 °C.

All DNA samples were initially screened for the presence of *Cryptosporidium* spp. via nested PCR amplification of an approximately 830 bp fragment of the *SSU* rRNA gene [22]. We performed PCR analysis for *Cryptosporidium* spp., conducting two technical replicates for each sample. Positive controls consisted of *C. bovis* DNA from cattle, and reagent-grade water served as the negative control. A subtyping analysis was performed on the positive samples using various genetic markers. The *gp60* gene was used for *C. parvum* [13], and four minisatellite/microsatellite loci (MS1, MS2, MS3, and MS16) were used for *C. andersoni* [14]. Positive amplicons were subsequently sequenced for species and subtype identification. All the primer sequences, annealing temperatures and expected amplicon sizes used in the present study are presented in Table S1 (Additional files: Table S1).

### 2.3. DNA Sequence and Phylogenetic Analysis

**Figure 2:**
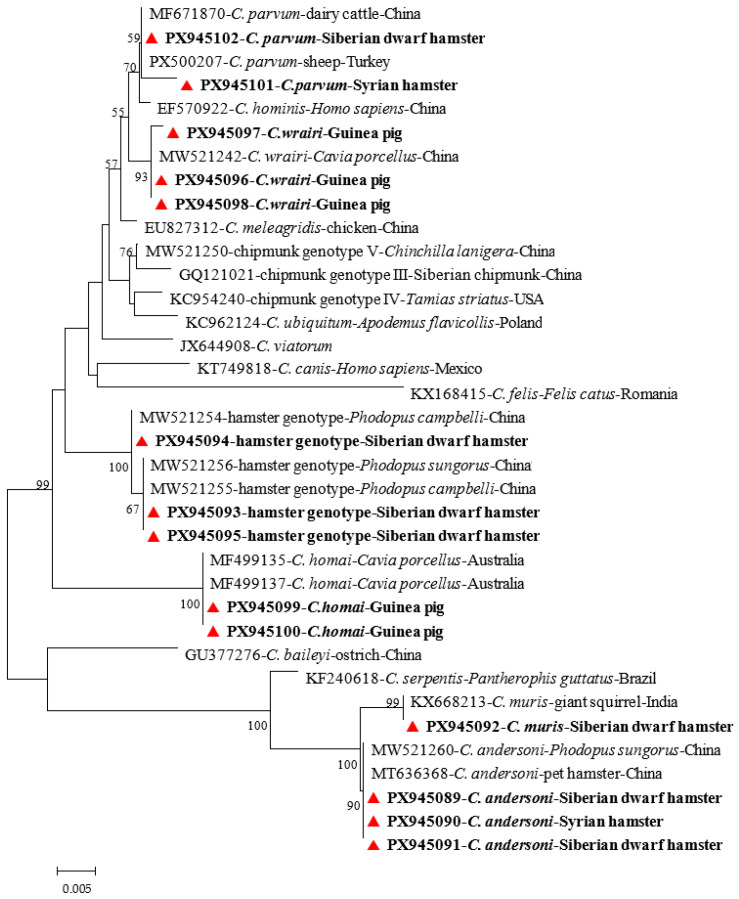
Maximum-likelihood phylogenetic tree of *Cryptosporidium* species based on *SSU* rRNA gene sequences. Sequences obtained in this study are indicated by solid red symbols.

All secondary PCR products that showed positive results on an agarose gel electrophoresis were submitted to Sangon Biotech (Shanghai, China) for bidirectional sequencing using an ABI 3730 sequencer (Applied Biosystems, Foster City, CA, USA). We assembled and manually edited the raw sequence chromatograms using ChromasPro 2.1.5.0 (http://technelysium.com.au/ChromasPro.html/, accessed on 15 January 2026) and BioEdit 7.1 software (http://thalljiscience.github.io, accessed on 6 February 2026), respectively. We performed multiple sequence alignments between the obtained sequences and the reference sequences were performed using ClustalX 2.1.5.0 (http://clustal.org/) to support species and subtype identification. Phylogenetic analysis was conducted using MEGA 7.0 software (http://www.megasoftware.net/) under the maximum likelihood method, with the general time-reversible model. Branch support was evaluated with 1000 bootstrap replicates, and only nodes with bootstrap values above 50% are indicated [23]. Phylogenetic trees were constructed based on the *SSU* rRNA gene sequences, as shown in Figure 2. The representative nucleotide sequences generated in this study have been deposited in GenBank under the accession numbers PX945089-PX945102, PX963158 and PX963159.

### 2.4. Statistical Analysis

The differences in *Cryptosporidium* spp. positivity rates among geographical regions, sampling locations, and host breeds were compared using the chi-square (*χ*^2^) test in SPSS 20.0 (IBM SPSS Inc., Chicago, IL, USA) and SAS 9.1 (SAS Institute Inc., Cary, NC, USA). Odds ratios (ORs) with 95% confidence intervals (CIs) were calculated using the Clopper-Pearson formula to quantify the strength of each potential risk factor in the univariable analysis. A two-tailed *p* < 0.05 was considered statistically significant. To illustrate the multilevel correspondences among rodent species, sampling regions, sampling locations, and *Cryptosporidium* species/genotypes, a Sankey diagram was generated using Origin 2026 software (https://www.originlab.com/index.aspx?go=Products/Origin/2026&pid=5478, accessed on 29 March 2026) in Figure S1 (Additional files: Figure S1). The diagram was constructed based on the number of positive samples identified in this study, with each node representing a categorical variable and the width of each flow proportional to the corresponding sample count.

## 3. Results

### 3.1. Occurrence of Cryptosporidium Species

*Cryptosporidium* spp. were detected in 131 (17.2%) of the 762 fecal samples collected from pet rodents across four cities in Yunnan Province (Table 2). Among these locations, the positivity rate in Qujing (10.5%) was lower than that in Zhaotong (21.0%, *χ*^2^ = 3.997, *p* = 0.046), but no statistically significant differences were observed when comparing Qujing with Yuxi (16.8%, *χ*^2^ = 1.969, *p* = 0.161) or Kunming (17.9%, *χ*^2^ = 3.079, *p* = 0.079). The occurrence in pet farms (18.6%) was slightly lower than in pet markets (19.5%, *χ*^2^ = 0.086, *p* = 0.769), but significantly higher than in pet shops (0%, *χ*^2^ = 14.28, *p* = 0.00016). The occurrence varied among the four pet rodent species, ranging from 0% to 18.7%. The occurrence in Syrian hamster (12.5%) was slightly lower than in Siberian dwarf hamster (17.3%, *χ*^2^ = 1.381, *p* = 0.240) and guinea pigs (18.7%, *χ*^2^ = 2.569, *p* = 0.109). No infection was detected in fancy rats (0/9). Two samples from a guinea pig farm in Yuxi tested positive for *Cryptosporidium* coinfection. Thus, the sampling location was significantly associated with *Cryptosporidium* infection. Region did not significantly influence infection occurrence, with the exception of the difference between Qujing and Zhaotong. Breed did not significantly influence infection occurrence.

### 3.2. Distribution of Cryptosporidium Species and Genotypes by Animal Species

Sequence and phylogenetic analyses of the *SSU* rRNA gene revealed six known *Cryptosporidium* species and genotypes (Table 3). The species and genotypes included *C. homai* (52/131), *C. wrairi* (30/131), *Cryptosporidium* sp. hamster genotype (25/131), *C. andersoni* (20/131), *C. parvum* (5/131) and *C. muris* (1/131). Among the six *Cryptosporidium* species and genotypes identified in the study, *C. homai* and *C. wrairi* were found in 52 and 30 guinea pigs, respectively, with two cases of coinfections. *Cryptosporidium* sp. hamster genotype and related sequences were found in 25 Siberian dwarf hamsters. *C. andersoni* was detected in 14 Syrian hamsters and six Siberian dwarf hamsters. The less common species, *C. parvum*, was detected in four Siberian dwarf hamsters and one Syrian hamster; *C. muris* was detected in one Siberian dwarf hamster. Therefore, most *Cryptosporidium* species exhibited a relatively specific association with a particular type of rodent.

Sequence analysis revealed that the following *Cryptosporidium* species/genotypes showed 100% homology with the reference sequences deposited in GenBank: *C. homai* was identical to GenBank sequence MF499137 from guinea pigs; *Cryptosporidium* sp. hamster genotype was identical to GenBank sequence MW521254 from hamsters; *C. andersoni* was identical to GenBank sequence MW521260 from hamsters; and *C. muris* was identical to GenBank sequence KX668213 from giant squirrels (Figure 2). Additionally, the nucleotide sequence of *C. wrairi* from guinea pigs had one nucleotide substitution compared to the GenBank sequence MW521242. Similarly, the nucleotide sequence of *C. parvum* from Siberian dwarf hamsters had two nucleotide substitutions compared to the GenBank sequence PX500207.

### 3.3. Subtyping of Cryptosporidium spp.

A total of 20 *C. andersoni*-positive samples were subtyped by MLST at four loci (MS1, MS2, MS3, and MS16), yielding one subtype, A3A4A2A2, of *C. andersoni* (*n* = 20). Sequence analysis revealed 100% homology with the reference sequences deposited in GenBank at each locus: MS1 (accession no. JF732836, derived from a hamster in Henan province, China), MS2 (accession no. MT780304, derived from beef cattle in Lanzhou province, China), MS3 (accession no. MK140455, derived from yak in Xizang province, China), and MS16 (accession no. JF732872, derived from a hamster in Henan province, China). No nucleotide polymorphisms or length variations were observed among the isolates at any of the four loci. The *gp60* gene was successfully sequenced from five *C. parvum*-positive samples. Aligning these sequences with reference sequences downloaded from GenBank identified one subtype. All *C. parvum* isolates from Siberian dwarf hamster and Syrian hamsters were identified as subtype IIdA15G1 (Table 3), showing 100% identity with the reference sequence KT964798 (derived from a dairy cow in Beijing, China).

## 4. Discussion

Our results suggest that *Cryptosporidium* spp. are common in pet rodents in Yunnan Province, with an overall positivity rate of 17.2% (131/762). This rate is comparable to the 21.8% reported in pet rodents from Zhengzhou [24], higher than that in Harbin (7.7%) [25], but lower than those in Guangzhou (36.9%) [26] and Sichuan (39.3%) [27]. Notably, the positivity rates observed in the present study were generally lower than those reported in previous studies for the same host species. In guinea pigs, the positivity rate (18.7%, 80/426) was substantially lower than the 52.3% reported in guinea pigs from Guangdong [26] and the 85.0% reported in another Chinese study [24]. Similarly, the combined positivity rate in hamsters (Siberian dwarf hamster and Syrian hamster) in Yunnan (17.3% and 12.5%, respectively) was lower than in Guangdong (68.4%) [26] and Sichuan (39.3%) [27]. These differences may be attributed to variations in geographic location, husbandry conditions, sample size, and study design [28].

The variation in positivity rates among species may be attributed to multiple factors, with animal management being a primary influence [26]. In this study, the positivity rate in rodents from pet markets and farms was significantly higher (19.5% and 18.6%, respectively) than in rodents from pet shops (0%). The higher positivity rates in rodents from pet markets and farms may be due to poor sanitary conditions and the co-housing of various susceptible species in shared environments [26]. In contrast, the cleaner environment in pet shops likely resulted in a lower positivity rate. This observation is supported by a previously report which found that poor breeding conditions and inadequate environmental hygiene were associated with more severe cryptosporidiosis [29].

This study revealed the high genetic diversity of *Cryptosporidium* spp. in pet rodents and identifying six known species and genotypes. *Cryptosporidium* infection appears to be associated with host-species. Infections were detected in guinea pigs, Siberian dwarf hamsters, and Syrian hamsters, but not in fancy rats, which is consistent with previous findings from Guangdong, China [26]. Notably, guinea pigs were exclusively infected with *C. wrairi* and *C. homai*, consistent with several earlier reports [26,29]. In contrast, hamsters were infected with a more diverse array of *Cryptosporidium* species and genotypes (*C. andersoni*, *Cryptosporidium* sp. hamster genotype, *C. parvum*, and *C. muris*), as reported in previous studies [24,26,27].

One *C. parvum* subtype (IIdA15G1), which is found in hamster, is a known zoonotic pathogen. *C. parvum* is one of the two most common species that cause human cryptosporidiosis and is of significant public health concern. Both the IIa and IId subtype families are recognized as zoonotic, with the IId subtypes being particularly prevalent among farm animals in China [16]. The IIdA15G1 subtype, identified in this study, has been frequently been reported in livestock across multiple Chinese provinces [30]. This subtype is also known to infect humans and has been associated with outbreaks of cryptosporidiosis in neonatal calves, providing evidence of its virulence in mammals [31]. *C. parvum* has been documented in at least 20 rodent species in China, including rats, mice, voles, and squirrels [26,32,33,34,35,36]. However, only two subtypes, IIdA15G1 and IIdA20G1, have been identified in rodents nationwide [26,37]. The presence of *C. parvum* subtype IIdA15G1 in pet hamsters indicates that these animals can transmit this subtype to humans, particularly in environments with frequent human–animal contact, such as households, pet shops, and breeding facilities [24]. Nevertheless, these findings underscore the need for increased awareness and further epidemiological studies to determine the role of pet rodents in transmitting *C. parvum* to humans.

The present study identified other zoonotic *Cryptosporidium* species, including *C. muris* and potentially *C. andersoni*. Both of these genetically related gastric parasites have been reported in human infections, although the zoonotic potential of *C. andersoni* remains under debate [29,38]. *C. muris* was detected in a Siberian dwarf hamster from a pet market. *C. muris* has a broad host range and has been reported in various rodents, other mammals, birds and in humans in developing countries [26,36]. In contrast, *C. andersoni* exhibited a notably higher prevalence, being identified in 20 hamsters sourced from pet markets. Subtyping analysis revealed that all 20 *C. andersoni* isolates belonged to a single subtype, A3A4A2A2. This subtype appears to be rare, having been previously reported only once in hamsters in China [24], whereas the other subtypes are most commonly identified in cattle [15,39]. The consistent detection of the A3A4A2A2 subtype in hamsters across different studies and time periods suggests a possible host adaptation of this particular *C. andersoni* subtype to hamsters, although its zoonotic potential remains to be further investigated.

The One Health approach emphasizes the interconnected health of humans, animals, and the environment [19]. The rising popularity of pet rodents, coupled with the close human–animal contact, underscores a growing public health concern. Our detection of the zoonotic parasite *C. parvum* IIdA15G1 in pet hamsters underscores these risks. Furthermore, our finding of significantly higher positivity rates in environments with poor hygiene and overcrowding identifies key modifiable risk factors. Addressing these risks requires adopting the One Health framework through collaboration among veterinarians, physicians, public health workers, and the pet industry [40].

## 5. Conclusions

This study revealed a moderate prevalence and genetic diversity of *Cryptosporidium* species among pet rodents in Yunnan Province. These findings provide important baseline data for a region where such epidemiological information was previously scarce. These findings significantly improve our understanding of the epidemiology of cryptosporidiosis within the expanding pet rodent trade and allow for a more accurate evaluation of its potential as a zoonotic threat. Six *Cryptosporidium* species/genotypes were identified, including the zoonotic species *C. parvum*, *C. muris*, and *C. andersoni*, as well as the host-adapted species *C. wrairi* and *C. homai*, and a hamster-specific genotype. Notably, *C. parvum* subtype IIdA15G1 and *C. andersoni* subtype A3A4A2A2 were detected in pet hamsters. The *C. parvum* subtype IIdA15G1 is a recognized zoonotic pathogen prevalent in livestock and humans in China, indicating that these popular pets carry *Cryptosporidium* subtypes with zoonotic potential. Significantly higher positivity rates in pet markets and farms compared to pet shops directly link transmission risk to suboptimal hygiene and high-density housing conditions. Therefore, the pet rodent industry must implement effective biosecurity and husbandry measures, coupled with public health education for owners and breeders, to prevent and control this disease.

## Figures and Tables

**Table 1:** Sampling of four pet rodent species from different locations in four cities of Yunnan, China.

Location	Region	No. of Samples				Total
		*Cavia porcellus* (Guinea Pig)	*Phodopus sungorus* (Siberian Dwarf Hamster)	*Mesocricetus auratus* (Syrian Hamster)	*Rattus norvegicus domestica* (Fancy Rat)	
Pet shops	Kunming	-	-	69	4	73
subtotal		-	-	69	4	73
Pet markets	Kunming	47	12	12	5	76
	Zhaotong	21	40	39	-	100
	Qujing	10	85	-	-	95
subtotal		78	137	51	5	271
Farms	Kunming	246	-	-	-	246
	Yuxi	102	70	-	-	172
subtotal		348	70	-	-	418
Total		426	207	120	9	762

**Table 2:** Prevalence and factors associated with *Cryptosporidium* spp. infection in pet rodents in Yunnan Province, China.

Variable	Category	No. Tested	No. Positive	Prevalence (%)	OR (95%, CI)	*p*-Value	Coinfection
Region	Kunming	395	71	17.9	1.86 (0.93–3.77)	0.244	-
	Yuxi	172	29	16.8	1.72 (0.80–3.71)		2
	Zhaotong	100	21	21.0	2.26 (1.00–5.09)		-
	Qujing	95	10	10.5	Reference		-
Location	Pet shops	73	0	0.0	-	<0.01	-
	Pet markets	271	53	19.5	1.06 (0.72–1.56)		-
	Breeding farms	418	78	18.6	Reference		2
Breed	*Cavia porcellus* (Guinea pig)	426	80	18.7	1.62 (0.89–2.93)	0.214	2
	*Phodopus sungorus* (Siberian dwarf hamster)	207	36	17.3	1.47 (0.77–2.82)		-
	*Mesocricetus auratus* (Syrian hamster)	120	15	12.5	Reference		-
	*Rattus norvegicus domestica* (Fancy Rat)	9	0	0.0	-		-
Total		762	131	17.2	-	-	2

No.: number; CI: confidence interval; OR: odds ratio.

**Table 3:** Species and subtypes identification of *Cryptosporidium* spp. from pet rodents in Yunnan Province, China.

Host	Location	No. of Samples	No. of Positive (%)	*Cryptosporidium* Species/ Genotype (No. of Samples)	*Cryptosporidium* Subtypes	Zoonotic risk of *Cryptosporidium* spp.
*Cavia porcellus* (Guinea pig)	Pet markets	78	8 (10.3%)	*C. wrairi* (7), *C. homai* (1)	-	Low-risk
	Breeding farms	348	72 (20.7%)	*C. homai* (51), *C. wrairi* (23) *	-	Low-risk
*Phodopus sungorus* (Siberian dwarf hamster)	Pet markets	137	30 (21.9%)	*Cryptosporidium* sp. hamster genotype (19), *C. andersoni* (6), *C. parvum* (4), *C. muris* (1)	*C. andersoni*-A3A4A2A2 (6) *C. parvum*-IIdA15G1 (4)	Risk
	Breeding farms	70	6 (8.6%)	*Cryptosporidium* sp. hamster genotype (6)	-	Low-risk
*Mesocricetus auratus* (Syrian hamster)	Pet markets	51	15 (29.4%)	*C. andersoni* (14), *C. parvum* (1)	*C. andersoni*-A3A4A2A2 (14) *C. parvum*-IIdA15G1 (1)	Risk
	Pet shops	69	0	-	-	-
*Rattus norvegicus domestica* (Fancy Rat)	Pet markets	5	0	-	-	-
	Pet shops	4	0	-	-	-
Total		762	131 (17.2%)	*C. homai* (52), *C. wrairi* (30), *Cryptosporidium* sp. hamster genotype (25), *C. andersoni* (20), *C. parvum* (5), *C. muris* (1) *	*C. andersoni*-A3A4A2A2 (20) *C. parvum*-IIdA15G1 (5)	

* The total number of *Cryptosporidium* species/genotypes identified is greater than the number of positive samples as a result of coinfections; Risk = includes known zoonotic species such as *C. parvum*; Low-risk = host-adapted species with rare reports in humans.

## Data Availability

The datasets presented in this study can be found in online repositories.

## Supplementary Materials

The following supporting information can be downloaded at: https://www.mdpi.com/article/10.3390/ani16081177/s1, Table S1: Oligonucleotide sequences and PCR conditions employed in this study. Figure S1. Sankey diagram demonstrating the associations among rodent species, sampling regions, sampling locations, and *Cryptosporidium* species/genotypes detected in this study.
