# Supplementary material for: Occurrence and Genetic Diversity of Cryptosporidium spp. in Pet Rodents from Yunnan, China: Identification of Zoonotic Subtypes in Hamsters
Source: Animals (Basel). 2026 Apr 12;16(8):1177. doi: 10.3390/ani16081177 (PMC13113912; doi:10.3390/ani16081177)
Supplement: Supplementary file 1 [file animals-16-01177-s001.zip › animals-4225206-supplementary.pdf]

# Supplementary Material

# Occurrence and genetic diversity of *Cryptosporidium* spp. in pet rodents from Yunnan, China: identification of zoonotic subtypes in hamsters

**Liujia Li [2,†], Xinjie Yang [1,†], Muhammad Sohail Sajid [3], Yongyi Wang [1], Ze Li [1], Qin Xie [1], Luyang Wang [1], Junjun He [1], Fengcai Zou [1,*] and Fanfan Shu [1,*]**

[1] The Yunnan Key Laboratory of Veterinary Etiological Biology, College of Veterinary Medicine, Yunnan Agricultural University, Kunming 650201, Yunnan Province, China; Yangxinjiee@sina.com (X.Y.); fan19829810607@163.com (Y.W.); Lz20041110@outlook.com (Z.L.); 15126606541@139.com (Q.X.); wly952659965@foxmail.com (L.W.); hejunjun617@163.com (J.H.)

[2] College of Agriculture and Biological Science, Dali University, Dali 671003, China; liliujia2007@163.com (L.L.)

[3] Department of Parasitology, University of Agriculture, Faisalabad 38000, Pakistan; drsohailuaf@uaf.edu.pk (M.S.)

* Correspondence: zfc1207@vip.163.com (F.Z.); shuff1227@163.com (F.S.)

† These authors contributed equally to this work

**Table S1.** PCR primers and expected amplicon size

| Usage | Gene | Primer Sequence (5' - 3') | Annealing temperatures (°C) | Amplicon Size (bp) |
|---|---|---|---|---|
| *Cryptosporidium* spp. identification | *SSU* rRNA | F1: TTC TAG AGC TAA TAC ATG CG<br>R1: CCC ATT TCC TTC GAA ACA GGA | 55 | 830 |
| | | F2: GGA AGG GTT GTA TTT ATT AGA TAA AG<br>R2: CTC ATA AGG TGC TGA AGG AGT A | 55 | |
| *Cryptosporidium parvum* subtype | *gp60* | F1: TTA CTC TCC GTT ATA GTC TCC<br>R1: GGA AGG AAC GAT GTA TCT GA | 52 | 800 |
| | | F2: TCC GCT GTA TTC TCA GCC<br>R2: GCA GAG GAA CCA GCA TC | 50 | |
| *Cryptosporidium andersoni* subtype | MS1 | F1: ACC ATC TAG AGA TAA CGA GCG A<br>R1: GAA TCA GAA GAT GAG CGA CAA | 55 | 550 |
| | | F2: CGT GAT AGT GGG TAT GAA TTG GAC A<br>R2: CGA CTG CGA TAC TCA CGT CCT | 55 | |
| | MS2 | F1: TTG CAA CTG TAC CTA AAT TAG TA<br>R1: GTG AGA CTT CTG GGG TCC TGA | 55 | 457 |
| | | F2: TCA TGA CGC GTC ATA CCA ACA<br>R2: ACT TAG ACA GTT CTA TGC TGA | 52 | |
| | MS3 | F1: AAC CAA GTG AAT CAC GAA CTT<br>R1: TCA AGT ACA GCA GTC TAT TGC TT | 55 | 536 |

| | | | | |
|---|---|---|---|---|
| | | F2: GCA ATA TCT TCG ACG ATC CCA<br>R2: ATG GGA ATA ATT CTT CAT CAT CAA | 55 | |
| | MS16 | F1: GAA GAG GTC GAA GTT AAG CTA<br>R1: GAC AAT CAT CTA AAT CGT GTT | 50 | 597 |
| | | F2: AAG TTT CAT CTA GGT ACA CTA AGA<br>R2: CAC TAC CTA ATC TCG TGT ACT T | 55 | |

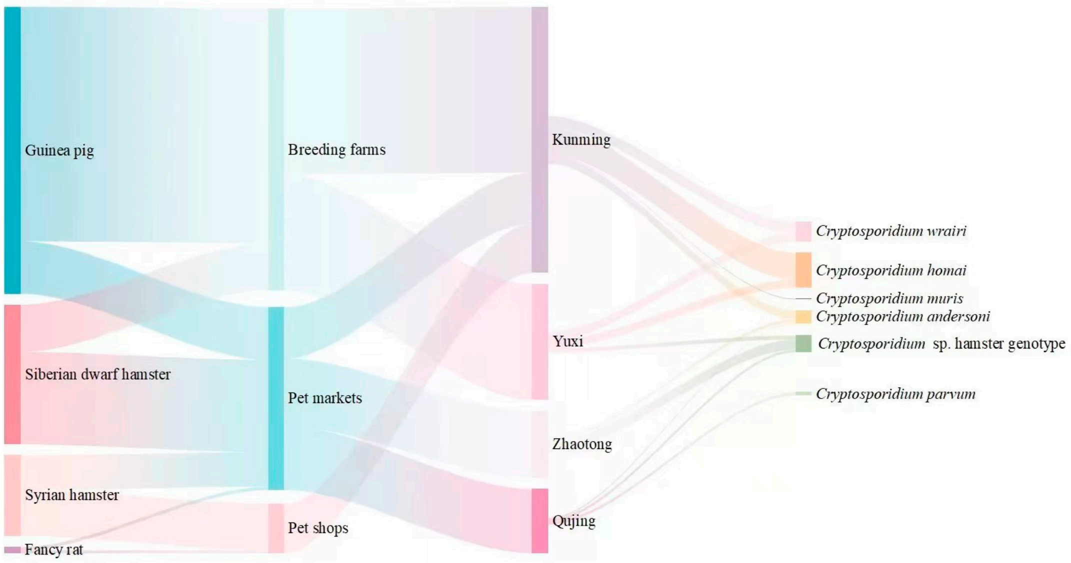


**Figure S1.** Sankey diagram demonstrating the associations among rodent species, sampling regions, sampling locations, and *Cryptosporidium* species/genotypes detected in this study.
